# *Ensete ventricosum*: A Multipurpose Crop against Hunger in Ethiopia

**DOI:** 10.1155/2020/6431849

**Published:** 2020-01-06

**Authors:** Getahun Yemata

**Affiliations:** Bahir Dar University, College of Science, Department of Biology, Mail-79, Bahir Dar, Ethiopia

## Abstract

*Ensete ventricosum* is a traditional multipurpose crop mainly used as a staple/co-staple food for over 20 million people in Ethiopia. Despite this, scientific information about the crop is scarce. Three types of food, viz., *Kocho* (fermented product from scraped pseudostem and grated corm), *Bulla* (dehydrated juice), and *Amicho* (boiled corm) can be prepared from enset. These products are particularly rich in carbohydrates, minerals, fibres, and phenolics, but poor in proteins. Such meals are usually served with meat and cheese to supplement proteins. As a food crop, it has useful attributes such as foods can be stored for long time, grows in wide range of environments, produces high yield per unit area, and tolerates drought. It has an irreplaceable role as a feed for animals. Enset starch is found to have higher or comparable quality to potato and maize starch and widely used as a tablet binder and disintegrant and also in pharmaceutical gelling, drug loading, and release processes. Moreover, enset shows high genetic diversity within a population which in turn renders resilience and food security against the ever-changing environmental factors and land use dynamics. Therefore, more research attention and funding should be given to magnify and make wider use of the crop.

## 1. Introduction

**Figure 1:**
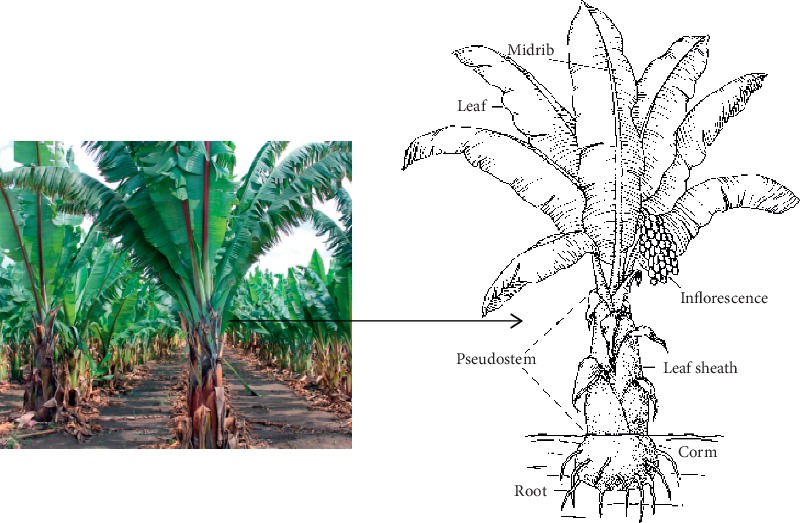
Enset plant and its parts.

*Ensete ventricosum* (Welw.) Cheesman is a perennial, monocarpic, herbaceous, and monocotyledonous crop [1] (Figure 1). It belongs to order Zingiberales, family Musaceae, and genus *Ensete* [1, 2]. *Musaceae* is a paleotropical family, forming the basal lineages of order Zingiberales. According to [1], *E. ventricosum* originated in Southeast Asia. Nevertheless, the wild representatives of the genus *Ensete* are found throughout Africa and Southern Asia [1]. In Africa, it occurs widely across the continent in the western, central, eastern, and southern parts [3]. Unlike its widespread distribution, enset has only ever been domesticated in Ethiopia. Even within Ethiopia, enset cultivation as a food crop is confined to a relatively small region of the southwest, in areas inhabited by speakers of Semitic, Cushitic, and Omotic languages [4].

*E. ventricosum* is a multipurpose crop commonly known by its vernacular name enset and widely cultivated in the south and southwestern Ethiopia. It is a traditional crop that makes up the enset-based farming system. It serves as a staple/co-staple food nourishing approximately more than 20 million people in the country [5, 6]. As a food crop, enset has several food security traits. First, the corm can be harvested at any time during the year and almost at any growth stage over a period of several years [7]. The corm is cooked and consumed, relieving hunger during periods of critical food shortage. Thus, the crop is considered as a field bank for food [8]. Second, enset foods can be stored for long periods [9, 10]. Third, compared to cereals, enset gives a high food yield per unit area. Enset growing regions of Ethiopia are well known for their high population density which may not be supported by any other type of land use and crop [11]. Due to the high population density, each farmer has very small land holding and yet the population rarely faces food shortage to eat and live because of the high productivity of enset. Fourth, enset grows in a wide range of environments from about 1200 to 3100 meters above sea level [5, 7]. This allows farmers to grow the plant in all parts of the country including areas not suitable for cereal cultivation. Fifth, enset is considered tolerant to drought, heavy rains, flooding, and other stress factors [9].

Moreover, research results suggest that “those populations who depend on enset have never suffered from famine, even during Ethiopia's tragic drought and famine prone decades of the 1970s and 1980s.” The crop has a potential role to contribute to ensure national food security [12, 13]. However, it was only after 1991 that attention was given to the crop whereby small research programs and experimental stations were established and endowed with operating funds and staff. The aim of this paper is to review research findings on enset, show its significant potential as a starch source for food and other applications, and magnify its visibility to the scientific community.

## 2. Diversity of *Ensete ventricosum*

Enset has high landrace (clone) diversity in Ethiopia. Yemataw et al. [14] have reported a total of 278 clones with distinct names from seven enset growing zones. Hadiya is the richest zone with a total of 59 clones followed by Kembata (43), Dawro (42), Wolaita (39), Gamo Gofa (34), Gurage (31), and Sidama (30). In another study, Tsegaye [8] has identified 146 different enset clones from Sidama, Wolaita, and Hadiya zones. Similarly, Negash [15] has recorded 146 different enset clones from four zones (65 from Kefa-Sheka, 30 from Sidama, 45 from Hadiya, and 6 from Wolaita). Moreover, Birmeta [16] has found 111 enset clones from nine enset growing areas of Ethiopia. The reports on the number of enset clones show high inconsistency which could be due to the fact that farmers distinguish landraces on the basis of phenotypic characteristics such as petiole, midrib and leaf sheath color, angle of leaf orientation, size and color of leaves, and circumference and length of the pseudostem [17]. The same landraces might have different vernacular names, and the same name can be given to different clones by the different ethnic or linguistic groups and agroecological zones [18]. It is naturally true that planting materials (clones) are exchanged between enset farmers of the same or different ethnic groups, and the vernacular names may be changed after long-term production of the clone, according to the farmer's own favorites and language [18]. The high phenotypic plasticity of enset clones further complicates identification based on morphological and physiological characteristics. Phenotypic plasticity is the capacity of a single genotype to express different phenotypes under different environmental conditions [19].

Birmeta [16] and Birmeta et al. [20] concluded that the current cultivated enset clones have been domesticated from a limited number of wild progenitors. However, subsequent gene flow between wild and cultivated ensets may have been inhibited by differences in modes of propagation and harvesting time. Nowadays, wild enset is clearly separated from cultivated enset and more closely related to the outgroup *Musa* spp. [21]. In cultivated enset, genetic diversity within populations is high [22]. Despite the large variation in agroecological conditions among enset-growing areas, amplified fragment length polymorphism (AFLP) studies revealed a total genetic variation of only 4.8% between regions and 95.2% within regions or populations [23]. This may be explained by the regular long-distance exchange of clones, gene flow, and the existence of substantial levels of phenotypic plasticity in enset due to changing weather and soil conditions [6, 23, 24]. Random amplified polymorphic DNA (RAPD) results have shown that the genetic diversity in cultivated enset in a particular area appears to be related to the extent of enset cultivation, the culture, and the distribution pattern of the different ethnic groups than geographical distance [16].

Furthermore, both increasing and decreasing number of landraces have been recorded over the years in the different enset-growing regions. This might be ascribed to the changing climate, food preferences, diseases, cultivation systems, and others [24]. For instance, farmers have increased the number of cultivated landraces to widen use values and secure availability of food and also to respond to annual climate fluctuations [24]. Two sources of variation are articulated in vegetatively propagated plants: somatic mutations and epigenetics [25]. Since plants have somatic and germline cells that appear together, somatic mutations can be transferred to germline through cell lineage from their descendants [25, 26]. In vegetatively propagated plants, somatic mutations accumulate through time and different branches inherit different mutations whereby the growing plant becomes a genetic mosaic resulting in unequal growth rates and genetically heterogeneous clonal descendants, which in turn contribute to intraindividual evolution [25, 26]. Moreover, the competition between cell lineages in a meristem that differ in somatic mutation leads to clonal selection or developmental selection and this filters mutations and results in a disproportionately high frequency of beneficial mutations being passed on to the next generation [25, 26]. Similarly, epigenetics is found to be important in evolution and ecology through its role in determining individual and population fitness especially in response to fluctuating environments. This is because environmental signals/cues influence epigenetic programming in which the information has the potential to pass to subsequent generations through gametes [27, 28]. The epigenetic information is transmitted either indirectly through epigenetic mark-induced behavioral/physiological changes or directly when the environmental factor affects the germline of the parent/interactions between the somatic cells and germline [27].

## 3. Enset Ecology and Cultivation

The genus *Ensete* is found throughout Africa and Southern Asia [1]. Enset flourishes in the cool tropics within a temperature range of 18 to 28°C. It requires a relative humidity of 60 to 80% and an annual rainfall of 1100 to 1500 mm. In Ethiopia, these conditions are met in the enset belt at altitudes between 1700 and 2450 meters above sea level (masl) [29]. However, farmers reproduce cultivated enset almost always vegetatively and expanded its cultivation area range from 1100 to 3000 masl [8]. Enset grows vigorously in various soil types as far as they have adequate nutrient and drainage, with pH values ranging from 5.6 to 7.3 and 2–3% organic matter [30]. Unlike banana, enset is more tolerant to wet conditions. It also competes well with grasses and other weeds to a degree that is exceptional in the *Musaceae* [29]. Enset can also survive short periods of frost and withstand prolonged drought periods [7]. It could survive even long time without enough rain and water [31]. Cultivated enset is an icon crop widely and densely distributed in the southern region, some part of Oromia, and small areas of southern and eastern parts of the Amhara Region [32]. According to the maximum entropy method for modeling species distribution, the primary hotspot areas of cultivated enset are found in Sidama, Gurage, Gedeo, Keffa, Sheka, Ari, Southern Omo, Benchi Maji, Arsi, and some part of Bale and West Shewa region [32].

Wild enset grows in several African countries, outside Ethiopia [33]. Earlier reports believe that wild enset has limited distribution in Ethiopia as compared to cultivated enset. Accordingly, wild enset grows mainly in the vicinity of Bonga city and in a smaller area around the Omo river, inhabiting places ranging from dense forests to open shrub land, or along riverbanks [16]. In these areas, they usually occur in a group of approximately 10–200 plants in a population [20]. On the other hand, Garedew et al. [34] found out wide distribution of the same plant in Sheka forest, Sheka zone [20]. In contrast, wild enset has been found highly spatially distributed in the Tigray and Benishangul Gumuz regions in addition to those regions where cultivated enset is found [32]. This report is corroborated by herbarium records that indicate the historical occurrence of wild enset in those areas [7]. The higher spatial distribution of wild enset is ascribed to the absence of cultural influence from the local community [32]. Wild enset reproduces naturally by seed [8] and grows better from 1036 to 3129 masl [32].

Enset farming is indigenous to Ethiopia and is a common feature of the farming systems in the south and southwestern parts of the country. It constitutes what is often termed as the “enset-based farming system.” In this system, enset is grown both in the homegarden and mainland as an intercrop and monocrop mode of crop cultivation [34]. Enset-growing areas are categorized into four subsystems based on the extent to which people depend on enset as a staple crop: (i) Sidama and Gurage where enset is grown as a staple food and main crop. (ii) The Gamo, Hadiya, Wolaita, Gedeo, and Ari people who use enset as a costaple crop with cereals and tuber crops [8]. (iii) Oromo farmers of southwestern Ethiopia who rely upon cereals as the most important crops and grow enset and root crops as secondary crops. Enset is grown largely for security reasons and eaten in the form of *Kocho* and *Amicho* (cooked corm). (iv) Sheko in southwestern Ethiopia growing root and cereal crops as primary and secondary dietary importance, respectively. Enset has a minor importance. The permanent enset-based farming system is closely linked to livestock. Dung is collected in the enclosures where the animals are kept at night and applied to enset plantations to enrich the soil in organic matter [29].

Naturally, enset can be multiplied by seed. Nonetheless, farmers reproduce enset exclusively by vegetative propagation using corm (Figure 1). This is because plants are harvested prior to seed set [35]. Enset-growing areas are characterized by diverse agroecologies and ethnic groups with distinct culture and cultivation practices. Thus, vegetative propagation of enset varies with these factors [36]. Accordingly, the traditional propagation processes include (1) uprooting of mother plants, (2) drying the corm, (3) splitting the corm, (4) wounding the apical meristem, (5) filling the corm with soil, manure, or gravel, and (6) planting, protection, and manuring of the propagated corm [36]. Several researches have been conducted so as to recommend economical and effective propagation practice. In this regard, Buke et al. [37] have found out the shortest emergence time and the highest number of enset suckers from a 3.5 kg corm piece taken from the corm apex and exposed to sunlight for a day. Similarly, Karlsson et al. [38] have got earlier emergence of sprouts from split parent corms than the whole and a corresponding number of suckers (sprouts) per parent corm have been obtained from pieces in which the parent corm is divided. The high number of vigorous suckers from split corms might be due to the removal of the apical dominance, leaving reasonable portions of the parent corm to sustain initiation, growth, and development of suckers [39]. During propagation, farmers usually apply manure on the soil surface, not directly on the corm at burial because they believe that direct contact causes rotting. However, the application of air-dried crushed manure thoroughly mixed with soil directly on the corm in the burial hole has produced higher number of suckers and subsequently biomass [38, 40]. Direct application might increase accessibility of nutrients and limit weed growth. Unlike the belief that watering causes rotting, it has resulted in the shortest time to emergence and vigorous growth of the suckers [38].

Each corm can yield on average 40–200 suckers depending on method of propagation, soil conditions, cultivar type, size and age of the parent corm, amount of rainfall, land preparation, and time of planting. Enset farms are established exclusively from vegetatively propagated suckers as farmers believe seedlings that develop from seeds are small in number and less vigorous [18]. Farmers use vegetative propagation to produce enset suckers in mass and maintain specific desirable traits of each clone/landrace [24]. It is also practiced for ease of propagation and useful to control gene flow from wild to cultivated enset [25]. Despite these virtues, vegetative propagation causes the accumulation and dissemination of pathogens [18], loss of some components of diversity, buildup of deleterious mutations, and higher competition between use of plant parts as propagules and use as food [25].

## 4. Enset Foods and Their Nutritive Value

Enset is a strategic food reserve that ideally suited to bridge food shortage, a common phenomenon in Ethiopia [8]. Outside Ethiopia, the inflorescence is eaten as an enjoyment in Malawi and the flower bud is consumed as a boiled vegetable in many parts of Southeast Asia [29]. The type and number of enset clones cultivated in southwestern Ethiopia vary from place to place. Large numbers of clones are cultivated for multiple purposes, under different site and climate requirements. In each area, different clones are grown for different purposes [24]. This shows that the different livelihood requirements of a farmer are partially or wholly covered by enset products. In areas where enset is an indigenous crop, it is the major source of human food supplemented with legumes and animal products [41].

Enset cultivation has been increasing over the years in terms of area coverage and production. Reports have shown that over 2 million tons of enset foods are produced each year. Enset crop loss is infrequent and production is high as compared to other crops [30]. Three main food items are prepared from enset: *Kocho* (fermented product from the corm and pseudostem, Figure 1), *Bulla* (dehydrated product of the juice from the decortication of the pseudostem and grating of corm), and *Amicho* is the stripped corm of younger plants of enset, boiled and consumed [41]. In 2017/18 cropping season, 127, 235, and 588 enset plants were planted from which 29, 307, and 635.04; 34, 782, and 944.88; and 1, 017, and 821.63 quintals of *Amicho*, *Kocho,* and *Bulla*, respectively, were obtained [30]. *Kocho* is the main enset food item. On average, one enset plant can produce 16.2 kg *Kocho*, which is equivalent to 417 tons/ha. The average annual *Kocho* demand of a person can be covered by 16 plants, 289 kg [42].

Enset is processed for consumption annually from November to March, and processing is an entirely women's task. The methods used in enset processing are more or less similar especially in the two steps (scraping of pseudostem and fermenting in a pit) in the various enset-growing areas [43]. The pseudostem (Figure 1) is decorticated and scraped to separate the pulp from the fibres. The corm (Figure 1) is scraped into pieces. The outputs are then chopped and added to pit. The starch deposits in these parts are then extracted through fermentation [44]. When Bulla is needed, the processed parts are squeezed and the resulting liquid is collected. This is dehydrated till forming a powder known as *Bulla* [43]. The fermentation period ranges from weeks to a few months and even to several years depending on environmental factors. The product is considered ready for consumption after 90 days from the initial processing day, but can also be kept for one or more years [29, 44]. *Kocho* can also be removed from the pits as needed and baked for use. Likewise, *Bulla* is white in color and relatively small in production quantity as compared to *Kocho*, but it still fetches a much higher price. Sometimes, corm chunks of certain enset clones are not fermented but eaten as a boiled form called *Amicho* [29].

In the traditional *Kocho* preparation process, a lot of physicochemical properties and aerobic mesophilic microorganism counts have shown decreasing trend and completely inhibited members of Enterobacteriaceae as the process proceeds and pH lowers down. Moreover, lactic acid bacteria and yeast have been identified as the major microorganisms accountable for the fermentation of *Kocho* [45]. Diverse species of yeast such as *Cryptococcus albidus* Var aerus*, Guilliermondella selenospora, Rhodotorula acheniorum*, *and Trichosporon beigelii* have been identified, of which 99, 98, and 86% of them are *Cryptococcus terreus*, *Candida zylandase,* and *Kluyveramyces delphensis,* respectively [46]. However, studies revealed that modifying the traditional *Kocho* processing lowers titratable acidity, increases pH, and alters the microbial diversity and density which subsequently improve the sensory detection of *Kocho* bread [47]. *Kocho* bread produced by the modified Gurage *Kocho* processing method has a better sensory preference than the one produced by the traditional method [47]. The findings of [48] have also shown that the length of fermentation time, amount of starter, and type of starter strain affects the sensory attributes of *Kocho*. Furthermore, blending *Kocho* (up to 40%) with white wheat and soybean flour has improved the nutritional profile including protein content and the congeniality of the most important bread sensory attributes such as color, texture, and taste [49].

Enset foods are very rich in carbohydrates and fibres. The quality of enset products depends on the age, type of clone, and method of processing [50]. The best quality *Kocho* is white in color and less fibrous produced when *Bulla* is not extracted [48]. Corms with few fibres are especially valued as they produce high quality *Kocho*. The lowest grade *Kocho* is darker and more fibrous and is an end product obtained after *Bulla* extraction [48]. Despite this perception, enset fibre has a very important dietary value. Fibres are the portions of plant foods that are resistant to digestion by the human digestive enzyme. Individuals with high intakes of dietary fibre appear to be at significantly lower risk for developing coronary heart disease, stroke, hypertension, diabetes, obesity, and certain gastrointestinal diseases such as gastroesophageal reflux disease, duodenal ulcer, diverticulitis, constipation, and hemorrhoids [51].

Several findings have suggested the potential protective role of dietary fibre to rectal and colon cancer [52, 53]. This happens through increase in fecal bulking and viscosity which subsequently reduce the contact time of carcinogens within the intestinal lumen, production of anticarcinogen short chain fatty acids, and increase bile acid deconjugation that further facilitates binding to carcinogens [52, 54]. Moreover, dietary fibres regulate many of the risk factors such as hypercholesterolemia, hypertension, obesity, and type II diabetes for coronary heart diseases. Dietary fibres prevent hypercholesterolemia by promoting the expression of major enzymes of *β*-oxidation and de novo lipogenesis and control the expression of some enzymes that minimizes cholesterol synthesis and augments excretion of cholesterol in bile [52, 55]. They also avert hypertension and obesity by slowing down carbohydrate absorption in the gut via increased viscosity [52].

## 5. Chemical Composition

The nutritive value of enset products is evaluated in terms of their chemical composition. The chemical composition of enset dry matter as a whole plant is 90.87% organic matter and 9.13% ash. The organic matter is again composed of 5.98% crude protein, 0.84% crude fat, 9.48% crude fibre, 74.57% soluble carbohydrates, and 60.62% starch [56]. The various enset parts have different contribution to the total dry weight, the highest being from the pseudostem and the lowest from the leaf lamina [57] (Figure 1). The unprocessed/raw pseudostem and corm are rich in soluble carbohydrates (80%) and starch (65%), but low in protein content (4%). Enset corm is found to possess 17 of the 20 amino acids with similar or higher concentration than potato. Enset leaves have 13% protein, among the highest in Ethiopia, 20% crude fibre, and 10% sugar [56]. Most enset parts are rich sources of minerals such as phosphorus, potassium, calcium, magnesium, iron, and manganese [41, 57, 58].

Enset production practices vary among the different ethnic groups. Maximum fresh weight of *Kocho* has been obtained in enset plants transplanted twice. Enset plants managed this way produce 54.1 kg/plant and 33 *t* ha ^−1^ y^−1^*Kocho*. This product has much higher edible dry weight and energy yields than any other crop cultivated in Ethiopia [11]. *Bulla* has higher energy (8.5 MJ/kg) than *Kocho* (6.5 MJ/kg). Both *Kocho* and *Bulla* are rich sources of carbohydrates. Nevertheless, *Koch*o has lower starch (75 g/100 g) than *Bulla* (89 g/100 g) on dry matter basis [59, 60]. The average daily intake of enset foods is 0.55 kg providing 68% of the total energy, the highest known, 20% of protein and 28% of iron. Both food products are poor in total fat and protein [59, 60].

According to [61], *Kocho* is composed of 3.6, 0.6, 0.5, 0.2, 0.1, 0.03, 0.02, 0.004, 0.009, 0.006, and 0.006 mg/g of K, Na, Ca, Mg, Fe, Zn, Cu, Mn, Ni, Cr, and Co, respectively. Similarly, *Bulla* is found to contain 0.8, 0.4, 0.4, 0.07, 0.05, 0.02, 0.003, 0.003, 0.004, ≤0.005, and 0.006 mg/g of K, Na, Ca, Mg, Fe, Zn, Cu, Mn, Ni, Cr, and Co, respectively. More or less similar concentrations of metals are reported by [60]. Higher concentrations of K followed by Na, Ca, and Mg have been reported for both foodstuffs. These foodstuffs are rich in Ca and Zn in comparison with other similar foodstuffs and have equivalent concentrations of Cu, Fe, and Mn [61]. In general, *Kocho* has higher concentration of most mineral nutrients than *Bulla*. Unlike the processed foodstuffs, the corm (unprocessed) is found to have higher concentration of Ca, Mg, K, Zn, and Fe [58]. Cd is found in the unprocessed enset corm, while Pb is below the detectable level of the methodology [58]. However, these metals have not been detected in processed enset products: *Kocho* and *Bulla* [61]. This shows that processing is useful in that it avoids harmful minerals in enset food products. Moreover, research reports have indicated that *Amich*o has the third highest ferric-reducing antioxidant power (FRAP) and total phenolics content next to teff and corn [41, 58]. This property of enset foods might also be related to the presence of phenylphenalenone type compounds. It is clear that the *in vitro* antioxidant capacity of teff and enset-based food ingredients is comparable with the commonly used staple carbohydrate sources such as wheat and corn [58]. Similarly, unfermented *Kocho* ranks third in total phenolic content while unfermented *Bulla* has the lowest [58].

Studies have revealed variations in chemical composition of enset plant parts and food products. This variation is attributed to the type of enset clone, harvesting age, management practice [59], spacing and frequency of transplanting, physical and chemical nature of the soil, method of cultivation, climatic conditions [23], and length of fermentation period [47]. According to [62], *Bulla* from the *Gewada* enset clone has a higher fat, fibre, carbohydrate, energy, and iron than other clones. Similarly, higher ash content and Ca concentration have been reported from *Bulla* of the *Yanbule* clone. *Bulla* extracted from *Zereta* and *Messena* clones is found to have higher protein content than the remaining clones [62]. Likewise, higher crude protein, crude fat, carbohydrate contents, higher crude fibre, total ash, and moisture contents have been reported in *Kinnare* and *Astare* clones of the Gurage zone, respectively [47]. The chemical composition of enset food items (*Kocho, Bulla*, and *Amicho*) also varies between cultivated and wild clones. Generally, cultivated enset clones have found to possess higher protein, fat, sugar, and minerals than the wild enset genotypes. Drought affects the chemical composition of enset parts. Extended drought has significantly reduced starch, crude protein, ash content, and potassium and phosphorus concentrations of the enset pseudostem and corm [63]. Talema and Fetene [64] have found nonsignificant variation in total nitrogen and crude protein content when the same clones are subjected to drought stress. This deviation might be caused by difference in sample processing and chemical determination methods. On the contrary, the crude fibre content, soluble sugars, and calcium levels have shown a significant increment in enset plant parts subjected to drought. Enset plants under drought have 2–4 folds greater soluble sugars than normally irrigated ones. The concentrations of crude fat content and/or magnesium remained unchanged [63].

Moreover, the composition enset foods show significant variation throughout the fermentation period. A significant reduction in total protein (15%), ash (16%), total carbohydrates (34%), starch (23%), soluble sugars (93%), reducing sugars (84%), and available carbohydrates (51%) has been reported after seven weeks of fermentation [65]. Contrary to this, the amounts of free amino acids and nonprotein nitrogen have increased by 6 and 1.6 fold, respectively. Organic acids such as lactic acid, isovaleric, and n-butyric acids followed by n-valeric acid and acetic acid are found to be the most abundant at the end of fermentation [65]. Moreover, the concentration of minerals such as iron (15%), phosphorus (29%), and calcium (51%) has been reduced through time. This might be related to the sharp lowering of pH (acidic pH). Against this finding, Bekele [62] has found increased protein, fat, ash, carbohydrate, total energy, mineral, and titratable acidity concentrations in the *Bulla* raw sample under prolonged fermentation.

## 6. Enset as Feed for Animals

Enset-growing areas are characterized by highest population density in Ethiopia, where the land holding of each farmer is very small [11]. In spite of the complementary relationship between crops and animals, as crop residues are used as animal feed and animals produce manure for crops in the enset-based farming system, most farmers lack grazing lands. In support of this, Menbere [66] has identified land and feed shortage and population pressure as major problems to the availability of animal feed in the area. The feed shortage has been tackled by using enset plant parts (leaf, pseudostem, and corm) both as basal feed and supplement for animals. Farmers have ranked nonconventional feeds such as enset as the best nutritious feed [67] and the acceptable level of consumption, the chemical composition, and the rate of degradability have found to be comparable or better than other typical animal feeds. Attributed to the high water content (85–90%), enset is especially important as an animal feed during the dry seasons when other feeds are scarce [56, 57].

In addition to its high water content, enset has a rich nutrient content. The leaf for instance contains high crude protein comparable to *Desmodium intortum* [57], fat, sugar, fibre, cellulose, hemicellulose, and lignin and lower soluble carbohydrates and starch comparable to the common local browse tree, *Sesbania sesban* [56]. The leaf has low dry matter degradability [57], but better than straw and banana and similar to stover and *Chloris guyana* [68]. Thus, sources of fermentable energy are necessary for the efficient utilization of the enset leaf as a feed [56, 57]. Unlike the enset leaf, the pseudostem and corm possess the highest soluble carbohydrates and starch [56]. The corm is also suitable for ensilage [56]. Furthermore, the results of several researches revealed that pseudostem, corm, and whole enset have higher dry matter degradability as compared to *Desmodium intortum,* wheat straw, *Chloris guyana*, setaria grass, elephant grass, and guatemala grass [57, 67, 68].

When examined in terms of nutrient composition and feed characteristics, greater total CP intakes, dry and organic matter digestibility coefficients, daily body weight gain, and feed conversion efficiency have been obtained in sheet when a basal diet of Rhodes grass hay is supplemented with different proportions of corm [69]. Based on their findings, Nurfeta and Eik [69] have recommended 129 g/DM/day of enset corm supplement as an alternative energy source to improve the productivity of sheep for small-scale farmers under enset livestock production systems. Unlike the leaf, treating the enset pseudostem with urea has brought a significant effect on dry matter intake [67, 68]. Similarly, the leaf has the highest neutral detergent fibre (NDF) and acid detergent fibre (ADF) as compared to other feeds [67, 68]. Opposite to this, research findings have shown lower NDF, ADF, and *in vitro* dry matter degradability (IVDMD) equivalent to the value reported for the best legume forages [67, 68]. The pseudostem has also the highest degradation rate (85.8%) after 48 hrs of incubation. Findings indicate that a complete animal feed can be formulated from enset as the leaf serves as a rich source of protein, and pseudostem and corm could be good sources of energy [57].

## 7. Industrial Applications

Starch is the major component of enset products. It has a lively and wider application in the pharmaceutical industries. Currently, it is used as a tablet binder and disintegrant and also in pharmaceutical gelling, drug loading, and release processes [70]. Studies have shown that enset starch has 29% amylose content, granule size, X-ray diffraction pattern, and gelatinization temperature that are comparable to potato starch, the commonly used material in pharmaceutical industries [71]. Nevertheless, enset starch has inferior quality than potato starch and superior quality than maize starch in terms of swelling powder, solubility, and peak viscosity [71]. Viscosity is a physical parameter referring to the level of resistance of a liquid to flow. This parameter affects the extrudability, spreadability, release of drug, and other physicochemical properties in gel formulations [72]. Research results of Gebre-Mariam and Nikolayev [73] and Beyene [74] have illustrated that enset starch can be used both as a tablet binder and disintegrant, possessing a better binding ability and less disintegrating power than potato starch.

The quality of enset starch used in the pharmaceutical industries can be improved and augmented by undertaking chemical, physical, or enzymatic modifications. For instance, cross-linking enset starch with microwave power and reaction time has produced a higher drug loading ratio and encapsulation efficiency than cross-linked cassava and potato starches [75]. This is because the cross-linking might form a dense enset starch matrix that could hold relatively large quantity of drugs relative to the less dense ones. Besides, the cross-linked enset starch matrix has released about 90% of the model drug, paracetamol, after 12 h showing its potential application in drug release sustaining pharmaceutical excipient [75]. Some poor properties of enset starch have not been improved by cross-linking. On the other hand, increased swelling powder and solubility and improved flow property and compactability have been obtained by acetylation and carboxymethylation substitution [72, 76]. Higher degrees of enset starch substitution have resulted in high tensile strength and longer disintegration time that subsequently make the material suitable for sustained-release drug formulations [72, 76].

Enset starch is also used as a gelling agent. The squeezed and dehydrated product of enset (*Bulla*) is used as a gelling agent substituting agar in *in vitro* propagation. According to Ayenew et al. [77], dried *Bulla* is used as a gelling agent, capable of producing an equivalent number of shoots, roots, leaves, shoot height, and associated fresh weight of plantlets in pineapple as agar. In Ethiopia, enset is basically cultivated for its starchy food. However, in the process, large amounts of biomass residues (byproducts) are produced mainly from pseudostem (leaf sheath) after being decorticated. Enset fibre is one such byproduct traditionally used to make sacks, bags, ropes, cordage, mats, and sieves in the rural areas [78]. It has an excellent structure and a strength equivalent to the fibre of abaca. Approximately, 600 tons of enset fibres have been sent to factories each year. This implies that enset fibre has a wide application in paper and pulp industries and also in construction as reinforcement in gypsum room decorations and panels [79].

Several studies have been done on morphological and chemical characterization of enset residue fibres. The morphological analysis reports have revealed longer fibre length, tinny cell wall thickness, large lumen diameter, thick fibre width [80], high tensile strength [79, 81], elongation at break, crystallinity index, and high thermal stability [81] compared to hard woods, agricultural residues, and bagasse. Chemically, enset residue fibre is found to possess the highest cellulose and smallest lignin and extractives as compared to the leaf fibre [80, 81], which is a plus quality for the fibre. Due to the positive correlation between fibre length and burst strength, tensile and tear strength and folding endurance have been estimated to produce strong paper [82]. The most important quality indicator to assess the suitability of any raw material for pulp and paper manufacturing is the Runkel ratio. This ratio is twice the ratio of wall thickness to lumen diameter. Any value below the standard (1) indicates acceptable pulp strength. The Runkel ratio of enset fibre is found to be 0.23 [80] which is very much lower than the standard. Lower Runkel ratio means thin fibre wall and larger fibre lumen width. The presence of thin fibre wall is appropriate for high quality, dense, and well-formed paper production [82].

The morphological and chemical analysis demonstrates that enset fibre fulfills fairly good properties for paper and pulp production except for its slenderness ratio [82]. The slenderness ratio of enset fibre is reported to be 58.4 [80], which is below the recommended value of 70. This implies that paper made from enset residue fibre could have low tear strength and thus may not be appropriate for wrapping and packaging purposes. Slenderness ratio also affects tensile and shattering strength of the fibre. Nevertheless, this property of enset residue fibre is compromised by the thin cell wall thickness [82]. In general, findings of several studies demonstrate that enset fibre has comparable properties to many of the world-class natural fibres such as abaca, flax, sisal, hemp, jute, kenaf, and ramie fibres [79–81, 83]. In spite of these qualities and potential, enset fibre has not been exploited for a variety of applications.

## 8. Medicinal Uses of Enset

Enset plays an important role in local medicine. Different enset clones are used for the same human and cattle ailments by the several ethnic groups. The leaves followed by the stem are the most frequently used parts for medicine [84]. The boiled *Amicho* and *Bulla* of the Tayo clone in Bonga [85], Ado, Genticha, Midasho, Gediwocho, and Kiticho clones in the Sidama zone [86] are mixed with milk and used to treat broken bone fractures and swellings with pus. The *Amicho* of the Choro clone in Bonga [85] and Asikala in Sidama [86] are formulated with butter and milk and given to women after delivery to stimulate placenta discharge. The *Amicho* of both enset clones supplemented with salt is given to dairy cows to cure similar ailments [85]. Similarly, the *Amicho* of Astara and Qibnar enset clones in Gedebano Gutazer Welene district, Gurage zone, has the same medicinal value as the abovementioned clones in Bonga and Sidama zones [84].

## 9. Conclusion

This review describes the versatile nature of *E. ventricosum* as a staple food and feed crop and important source of materials for traditional and industrial applications. Enset shows high genetic diversity within a population than between regions. This might be ascribed to the long-distance exchange of clones, gene flow, and high level of phenotypic plasticity. As a food crop, enset has several desirable traits such as the corm can be harvested at any time, enset foods can be stored for long periods, produces high yield per unit area, grows in a wide environmental range, and tolerant to drought, flooding, and high temperature. Three food items, namely, *Kocho*, *Bulla*, and *Amicho* are produced from the enset pseudostem and corm. The different ethnic groups have more or less similar food processing techniques. Enset foods are extremely rich in carbohydrates, minerals, and fibres, but poor in proteins. The leaf and pseudostem are also excellent feeds to animals, more importantly during the dry season when other sources are absent. Furthermore, enset starch has been used as a tablet binder, disintegrant, and gelling agent in the pharmaceutical industries. However, the research efforts so far did not bring any significant improvement in exploiting the crop to the maximum potential. Therefore, more research towards magnifying the desirable traits and wider expansion and utilization of the crop throughout the country is recommended.

## References

[1] Kress W. J., Prince L. M., Hahn W. J., Zimmer E. A. (2001). Unraveling the evolutionary radiation of the families of the *Zingiberales* using morphological and molecular evidence. *Systematic Biology*.

[2] Simpson M. G. (2006). *Plant Systematics*.

[3] Jones D. R., Jones D. R. (2000). Introduction to banana, abaca and enset. *Diseases of Banana, Abaca and Enset*.

[4] Blench R., Amha A., Mous M., Sava G. (2007). Enset culture and its history in highland Ethiopia. *Omotic and Cushitic Language Studies*.

[5] Addis T., Azerefegne F., Blomme G., Kanaujia K. (2008). Biology of the enset root mealybug cataenococcus ensete and its geographical distribution in southern Ethiopia. *Journal of Applied Biosciences*.

[6] Magule T., Tesfaye B., Catellani M., Pe M. E. (2014). Indigenous knowledge, use and on-farm management of enset (*Ensete ventricosum* (Welw.) Cheesman) diversity in Wolaita, Southern Ethiopia. *Journal of Ethnobiology and Ethnomedicine*.

[7] Borrell J. S., Biswas M. K., Goodwin M. (2019). Enset in Ethiopia: a poorly characterized but resilient starch staple. *Annals of Botany*.

[8] Tsegaye A. (2002). On indigenous production, genetic diversity and crop ecology of enset (*Ensete ventricosum* (Welvv.) Cheesman).

[9] Fekadu D. (2009). Characterizing farming practices from three regions of Ethiopia on which enset (*Ensete ventricosum*) is widely profited as a multipurpose crop plant. *Livestock Research for Rural Development*.

[10] Ayalew A., Yeshitila M. (2011). The response of enset (*Ensete ventricosum* (Welw.) Cheesman) production to rate and frequency of N and P nutrients application at Areka, Southern Ethiopia. *Innovative Systems Design and Engineering*.

[11] Tsegaye A., Struik P. C. (2001). Enset (*Ensete ventricosum* (Welw.) Cheesman) kocho yield under different crop establishment methods as compared to yields of other carbohydrate-rich food crops. *NJAS—Wageningen Journal of Life Sciences*.

[12] Tsegaye A., Struik P. C. (2002). Analysis of enset (*Ensete ventricosum*) indigenous production methods and farm-based biodiversity in major enset-growing regions of southern Ethiopia. *Experimental Agriculture*.

[13] Negash A., Niehof A. (2004). The significance of enset culture and biodiversity for rural household food and livelihood security in southwestern Ethiopia. *Agriculture and Human Values*.

[14] Yemataw Z., Mohamed H., Diro M., Addis T., Blomme G. (2014). Ethnic-based diversity and distribution of enset (*Ensete ventricosum*) clones in southern Ethiopia. *Journal of Ecology and the Natural Environment*.

[15] Negash A. (2001). Diversity and conservation of enset (*Ensete ventricosum* Welw. Cheesman) and its relation to household food and livelihood security in South-Western Ethiopia.

[16] Birmeta G. (2004). Genetic variability and biotechnological studies for the conservation and improvement of *Ensete ventricosum*.

[17] Shambulo A., Gecho Y., Tora M. (2012). Diversity, challenges and potentials of enset (*Ensete ventricosum*) production: in case of offa woreda, Wolaita zone, southern Ethiopia. *Food Science and Quality Management*.

[18] Yemataw Z., Lingua::EN::Titlecase A., Bekele A. (2018). A review of enset (*Ensete ventricosum* (Welw.) Cheesman) diversity and its use in Ethiopia. *Fruits*.

[19] Gianoli E., Valladares F. (2012). Studying phenotypic plasticity: the advantages of a broad approach. *Biological Journal of the Linnean Society*.

[20] Birmeta G., Nybom H., Bekele E. (2004). Distinction between wild and cultivated enset (*Ensete ventricosum*) gene pools in Ethiopia using RAPD markers. *Hereditas*.

[21] Magule T., Tesfaye B., Pagnotta M. A., Pe M. E., Catellani M. (2015). Development of SSR markers and genetic diversity analysis in enset (*Ensete ventricosum* (Welw.) Cheesman), an orphan food security crop from Southern Ethiopia. *BMC Genetics*.

[22] Chombe D., Bekele E. (2011). Analysis of genetic diversity among cultivated enset (*Ensete ventricosum*) populations from Essera and Kefficho, southwestern part of Ethiopia using inter simple sequence repeats (ISSRs) marker. *African Journal of Biotechnology*.

[23] Negash A., Tsegaye A., Van Treuren R., Visser B. (2002). AFLP analysis of enset clonal diversity in south and southwestern Ethiopia for conservation. *Crop Science*.

[24] Zippel K., Hube B., Sinclair B. J., Lampe K. H. (2005). Diversity over time and space in enset landraces (*Ensete ventricosum*) in Ethiopia. *African Biodiversity*.

[25] McKey D., Elias M., Pujol B., Duputié A. (2010). The evolutionary ecology of clonally propagated domesticated plants. *New Phytologist*.

[26] Cruzan M. B., Streisfeld M. A., Schwoch J. A. (2018). phenotypic effects of somatic mutations accumulating during vegetative growth. https://www.biorxiv.org/content/10.1101/392175v1.

[27] Duncan E. J., Gluckman P. D., Dearden P. K. (2014). Epigenetics, plasticity, and evolution: how do we link epigenetic change to phenotype?. *Journal of Experimental Zoology Part B: Molecular and Developmental Evolution*.

[28] John R. M., Rougeulle C. (2018). Developmental epigenetics: phenotype and the flexible epigenome. *Frontiers in Cell and Developmental Biology*.

[29] Deckers J., Tessera M., Alemu K., Abate T., Swennen R., Raemaekers R. H. (2001). Enset (*Ensete ventricosum* (welw.) cheesman. *Crop Production in Tropical Africa*.

[30] Workneh S., Neela S. (2019). A review on nutritional profile of the food from enset: a staple diet for more than 25 per cent population in Ethiopia. *Nutrition & Food Science*.

[31] Tamire C. (2017). Diversity and resilient varieties of enset for climate change adaptation: the case of different agro-ecological zones of Hadiya, southern Ethiopia. *Journal of Earth Science & Climatic Change*.

[32] Awoke M., Tesfaw B., Demissew S. (2019). Spatial characterization and distribution modelling of *Ensete ventricosum* (wild and cultivated) in Ethiopia. *Geocarto International*.

[33] Acero M., Mukasa S. B., Baguma Y. (2018). Morphotypes, distribution and uses of false banana in Uganda. *African Crop Science Journal*.

[34] Garedew B., Ayiza A., Haile B., Kasaye H. (2017). Indigenous knowledge of enset (*Ensete ventricosum* (Welw.) Cheesman) cultivation and management practice by Shekicho people, southwest Ethiopia. *Journal of Plant Sciences*.

[35] Karlsson L. M., Tamado T., Lagibo A., Mikias Y. (2013). Early growth and development of *Ensete ventricosum* (Musaceae) seedlings. *Journal of Plant Sciences*.

[36] Zippel K., Ludders P. (2004). *Propagation of Enset (Ensete ventricosum) Among Different Climate and Cultural Conditions*.

[37] Buke T., Tesfaye B., Kefale D. (2016). Studies on conventional vegetative propagation of enset (*Ensete ventricosum* (Welw.) Cheesman). *International Journal of Research And Innovations in Earth Sciences*.

[38] Karlsson L. M., Dalbato A. L., Tamado T., Mikias Y. (2015). Effect of cultivar, traditional corm pre-treatment and watering on sprouting and early growth of enset (*Ensete ventricosum*) suckers. *Experimental Agriculture*.

[39] Diro M., Gebremariam S., Zelleke A., Van Staden J. (2002). Growth of enset (*Ensete ventricosum*) suckers under different horticultural practices. *South African Journal of Botany*.

[40] Bosha A., Dalbato A. L., Tana T., Mohammed W., Tesfaye B., Karlsson L. M. (2019). Effect of manure amount and improved application technique at corm burial on the propagation of enset (*Ensete ventricosum*) suckers. *Folia Horticulturae*.

[41] Forsido S. F., Rupasinghe H. P. V., Astatkie T. (2013). Antioxidant capacity, total phenolics and nutritional content in selected Ethiopian staple food ingredients. *International Journal of Food Sciences and Nutrition*.

[42] Sahle M., Yeshitela K., Osamu S. (2018). Mapping the supply and demand of enset crop to improve food security in Southern Ethiopia. *Agronomy for Sustainable Development*.

[43] Fekadu A., Vandeweyer D., Teffera F., Vancampenhout K., Woldesenbet F., Van Campenhout L. (2018). Effect of fermentation system on the physicochemical and microbial community dynamics during enset (*Ensete ventricosum*) fermentation. *Journal of Applied Microbiology*.

[44] Brihanu Z. T., Gizaw B. (2015). Community indigenous knowledge on traditional fermented enset product preparation and utilization practice in Gedeo zone. *Journal of Biodiversity and Environmental Sciences*.

[45] Habte T., Abegaz K., Negera E. (2014). The microbiology of *Kocho*: an Ethiopian traditionally fermented food from enset (*Ensete ventricosum)*. *International Journal of Life Sciences*.

[46] Gizaw B., Tsegaye Z., Tilahun B. (2016). Isolation and characterization of yeast species from *Ensete ventricosum* product; *Kocho* and *Bulla* collected from Angacha district. *International Journal of Advanced Biological and Biomedical Research*.

[47] Temesgen M. (2013). Improving the indigenous processing of *Kocho*; an Ethiopian traditional fermented food. *Journal of Nutrition & Food Sciences*.

[48] Weldemichael H., Admassu S., Alemu M. (2018). Optimization of enset fermentation in the production of Kocho using response surface methodology. *Acta Universitatis Cibiniensis. Series E: Food Technology*.

[49] Gebremeskel A., Abegaz K., Ahmed M. (2018). The Physico-chemical composition and sensorial quality of *Kocho* bread blended with wheat (*Triticum aestivum*) and soybean (*Glycine Max*) flour. *CPQ Nutrition*.

[50] Karssa T., Papini A. (2018). Effect of clonal variation on quality of *Kocho*, traditional fermented food from enset (*Ensete ventricosum*), Musaceae. *International Journal of Food Science and Nutrition Engineering*.

[51] Ötles S. (2014). Health effects of dietary fiber. *Acta Scientiarum Polonorum Technologia Alimentaria*.

[52] Ozgoz M. M., Miller M. J., Freund G. G. (2012). The health benefits of dietary fiber: beyond the usual suspects of type 2 diabetes mellitus, cardiovascular disease and colon cancer. *Metabolism*.

[53] Gianfredi V., Nucci D., Salvatori T. (2019). Rectal Cancer: 20% risk reduction thanks to dietary fibre intake, Systematic review and meta-analysis. *Nutrients*.

[54] Zeng H., Lazarova D. L., Bordonaro M. (2014). Mechanisms linking dietary fiber, gut microbiota and colon cancer prevention. *World Journal of Gastrointestinal Oncology*.

[55] Lattimer J. M., Haub M. D. (2010). Effects of dietary fiber and its components on metabolic health. *Nutrients*.

[56] Mohammed B., Martin G., Laila M. K. (2013). Nutritive values of the drought tolerant food and fodder crop enset. *African Journal of Agricultural Research*.

[57] Nurfeta A., Tolera A., Eik L. O., Sundstøl F., Sundstol F. (2009). Feeding value of enset (*Ensete ventricosum*), Desmodium intortumhay and untreated or urea and calcium oxide treated wheat straw for sheep. *Journal of Animal Physiology and Animal Nutrition*.

[58] Debebe A., Chandravanshi B. S., Wondimu T. (2012). Metallic nutrients in enset (*Ensete ventricosum*) corm cultivated in Wolliso and Wolkite towns in Ethiopia. *SINET: Ethiopian Journal of Science*.

[59] Daba T., Shigeta M. (2016). Enset (*Ensete ventricosum*) production in Ethiopia: its nutritional and socio-cultural values. *Agriculture and Food Sciences Research*.

[60] Chaka A. (2019). Value chain and nutritional analyses of *Warqe* food products in relation to post-harvest losses.

[61] Atlabachew M., Chandravanshi B. S. (2008). Levels of major, minor and trace elements in commercially available enset (*Ensete ventricosum* (Welw.), Cheesman) food products (Kocho and Bulla) in Ethiopia. *Journal of Food Composition and Analysis*.

[62] Bekele H. (2015). Study on the Effect of Enset (*Ensete ventricosum* (Welw), Cheesman) Variety and Fermentation on Nutritional Composition, Anti-nutritional Factors, and Physicochemical Characteristics and Functional Property of Bulla: From Wolaita, Ethiopia.

[63] Zewdie S., Olsson M., Fetene M. (2008). Effect of drought/irrigation on proximate composition and carbohydrate content of two enset (*Ensete ventricosum* (Welw.) Cheesman) clones. *SINET: Ethiopian Journal of Science*.

[64] Talema A., Fetene M. (2014). Effect of seasonal irrigation on nitrogen and crude protein content of enset (E*nsete ventricosum* (Welw.) Cheesman). *Research Journal of Agricultural and Environmental Management*.

[65] Urga K., Fite A., Biratu E. (1997). Natural fermentation of enset *(Ensete ventricosum*) for the production of *kocho*. *Ethiopian Journal of Health Development*.

[66] Menbere S. (2014). Livestock feeds and feeding system in enset (*Ensete ventricosum*) dominated mixed farming system of southern Ethiopia. *Journal of Animal and Feed Research*.

[67] Gemiyo D. (2015). Evaluation of major feed resources in crop-livestock mixed farming systems, southern Ethiopia: indigenous knowledge versus laboratory analysis results. *Journal of Agriculture and Rural Development in the Tropics and Subtropics*.

[68] Fekadu D., Ledin I. (1997). Weight and chemical composition of the plant parts of enset (*Ensete ventricosum)* and the intake and degradability of enset by cattle. *Livestock Production Science*.

[69] Nurfeta A., Eik L. O. (2014). Assessment of different levels of enset (*Ensete ventricosum*) corm as an energy supplement in sheep fed a basal diet of Rhodes grass hay. *Tropical Animal Health and Production*.

[70] Tesfaye A., Girma A. (2017). Phytochemistry, pharmacology and nutraceutical potential of enset (*Ensete ventricosum*). *African Journal of Basic & Applied Sciences*.

[71] Gebre-Mariam T., Abeba A., Schmidt P. C. (1996). Isolation and physico-chemical properties of enset starch. *Starch—Starke*.

[72] Gabriel T., Belete A., Gebre-Mariam T. (2013). Preparation and evaluation of carboxymethyl enset and cassava starches as pharmaceutical gelling agents. *Journal of Drug Delivery and Therapeutics*.

[73] Gebre-Mariam T., Nikolayev A. S. (1993). Evaluation of starch obtained from *Ensete ventricosum* as a binder and disintegrant for compressed tablets. *Journal of Pharmacy and Pharmacology*.

[74] Beyene B. (2015). Evaluation of pregelatinized enset (*Ensete ventricosum*) starch as a tablet disintegrant in enteric coated acetyl salicylic acid tablet.

[75] Abrha S., Belete A., Gebre-Mariam T. (2011). Comparative study of the physicochemical, drug loading and releasing properties of cross-linked cassava, enset and potato starches. *Ethiopian Pharmaceutical Journal*.

[76] Nigussu E., Belete A., Gebre-Mariam T. (2013). Acetylation and characterization of enset starch and evaluation of its direct compression and drug release sustaining properties. *International Journal of Pharmaceutical Sciences and Research*.

[77] Ayenew B., Mengesha A., Tadesse T., Gebre-Mariam E. (2012). *Ensete ventricosum (*Welw*.)* Cheesman: a cheap and alternative gelling agent for pineapple (*Ananas comosus* var. smooth cayenne) *in vitro* propagation. *Journal of Microbiology, Biotechnology and Food Sciences*.

[78] Brandt S. A., Spring A., Hiebsch C. (1997). *The “Tree against Hunger” Enset-Based Agricultural Systems in Ethiopia*.

[79] Blomme G., Yemataw Z. (2018). Assessing enset fibre yield and quality for a wide range of enset (*Ensete ventricosum* (Welw.) Cheesman) landraces in Ethiopia. *Fruits*.

[80] Berhanu H., Kiflie Z., Feleke S., Yimam A. (2016). Chemical and morphological analysis of enset *(Ensete ventricosum)* fibre, leaf, and pseudostem. *Lignocellulose*.

[81] Teli M. D., Terega J. M. (2017). Chemical, physical and thermal characterization of *Ensete ventricosum* plant fibre. *International Journal of Engineering Research and Technology*.

[82] Ogunjobi K. M., Adetogun A. C., Omole A. O. (2014). Assessment of variation in the fibre characteristics of the wood of *Vitex doniana* sweet and its suitability for paper production. *Journal of Research in Forestry, Wildlife and Environment*.

[83] Berhanu H., Kiflie Z., Miranda I. (2018). Characterization of crop residues from false banana/*Ensete ventricosum*/in Ethiopia in view of a full-resource valorization. *PLoS One*.

[84] Abdella K. (2016). Traditional medicinal use of *Ensete ventricosum* (Welw.) cheesman in gedebano gutazer Welene district, Gurage zone, SNNP region, Ethiopia.

[85] Tesfaye Y., Kebede F. (2006). Diversity and cultural use of Enset (*Ensete ventricosum* (Welw.) Cheesman) in Bonga *in situ* conservation site, Ethiopia. *Ethnobotany Research and Applications*.

[86] Assefa A. S., Fitamo D. (2016). The ethnobotanical study and distribution patterns of enset landraces (*Ensete ventricosum* (welw) cheesman) in aleta chuko district, Sidama zone, south nation nationality people and regional state, Ethiopia. *Research & Reviews: Research Journal of Biology*.

